# Motivation to train during a pandemic: The role of fitness resources, mental health, and motivational profiles among student-athletes in team sports

**DOI:** 10.3389/fspor.2022.954086

**Published:** 2022-09-09

**Authors:** Rebecca J. Purc-Stephenson, Thomas M. J. Zimmerman, Rachel Edwards

**Affiliations:** Department of Social Sciences, University of Alberta, Camrose, AB, Canada

**Keywords:** COVID-19, motivation, mental health, return to sport, students

## Abstract

The sporting season across post-secondary institutions was canceled in March 2020 due to COVID-19, and student-athletes had to maintain their training at home. It is unclear what personal and contextual factors facilitated student-athletes' ability to maintain their training routines at home when social distancing and lockdown (SD/L) policies were put in place. Our cross-sectional study of 433 student-athletes examined (a) how athletes adapted their training, (b) what training barriers they experienced, (c) whether motivational profiles were associated with differences in training behaviors and mental health, and (d) what variables predicted athletes' motivation to train during this prolonged offseason. Student-athletes across Canada were recruited to complete an online survey between August and September 2020. Results showed that athletes significantly reduced their training load and intensity, with approximately 25% exercising two or fewer days a week. Barriers to training included limited access to fitness resources and equipment, having inconsistent training schedules, and experiencing emotional distractions, with some of these barriers more common among female athletes than male athletes. For motivation profiles, athletes with higher levels of intrinsic motivation tended to maintain the intensity of their workouts and experienced lower mood disturbance. A hierarchical multiple regression revealed that being male, being younger, having higher levels of intrinsic and introjected motivation, having access to fitness resources, maintaining a steady training schedule, having fewer emotional distractions, and lower mood disturbance were significant predictors to being motivated to train during the pandemic. We discuss strategies coaches and trainers can implement to best support their student-athletes.

## Introduction

In Canada, more than 20,000 students participate in an organized sport at a university or college each year (Canadian Collegiate Athletic Association, 2020). However, the post-secondary sporting season abruptly ended in March 2020 as health authorities enacted social distancing and lockdown (SD/L) policies to slow spread of COVID-19 (Government of Canada, 2020; World Health Organization, 2021), which attacks the respiratory system and is most frequently spread when people are in close contact with others who are infected (Centers for Disease Control and Prevention, 2020).

As the post-secondary sporting season was canceled, student-athletes entered what became a prolonged offseason and had to maintain their training at home. However, many experienced barriers to training as parks and fitness centers were also closed (Pillay et al., 2020; Facer-Childs et al., 2021). Further, the unexpected and sudden change in daily routine, home confinement, quarantine, and the uncertainty of when the sporting season would resume contributed to athletes' distress (Leguizamo et al., 2021; Rubio et al., 2021), depression (Batalla-Gavalda et al., 2021; Facer-Childs et al., 2021), and anxiety (González-Hernández et al., 2021; McGuine et al., 2021). Mental distress has been associated with an increased risk of injury and lower athletic performance (Wiese-Bjornstal, 2019). Despite the struggles, the motivational profiles of some athletes may have helped them adapt during this time (Ruffault et al., 2020; Morbée et al., 2021). To date, few studies have examined the relationship between training behavior, mental health, and motivation among athletes during the pandemic. Thus, we investigated whether access to fitness resources, mental health, and motivational profiles predicted student-athletes' motivation to train during this prolonged offseason.

### Training and the impact of COVID-19

Athletes continuously exercise and regulate their training loads to enhance their physical and mental performance (Facer-Childs et al., 2021). However, decreasing training loads during the pandemic was common among elite athletes (Pillay et al., 2020; Facer-Childs et al., 2021). For example, Pillay et al. (2020) surveyed elite athletes in South Africa and found that 75% reduced their training loads and intensity during lockdown in April 2020, and the majority engaged in sedentary behavior such as watching television to fill their free time. Reducing training loads and increasing sedentary behavior can negatively impact athletic performance and increase the risk of injury (Ciddi and Yazgan, 2020; Girardi et al., 2020; Saffar and Kordi, 2020). Furthermore, it was common for athletes to train alone or unsupervised during the pandemic. For example, Washif et al. (2021) surveyed over 12,000 athletes across 142 countries and found that 80% reported training alone and focusing on general health instead of sport-specific training due to a lack of space or equipment. Indeed, access to training resources was a significant barrier for student-athletes to maintain their training (National Collegiate Athletic Association Research, 2020), and it was even more problematic for athletes that had sport-specific equipment needs such as a pool, a court, or a weight rack (Latella et al., 2018; Shepherd et al., 2021). To date, there is limited understanding how Canadian student-athletes adapted their training during the pandemic.

### Mental health and the impact of COVID-19

Aside from training, athletes experience a unique set of stressors that increases their risk of poor mental health. A systematic review by Rice et al. (2016) found that elite athletes often experience poor-quality relationships, travel demands, stress due to competitive failure and retirement from sport. Student-athletes face additional stressors of having to balance athletic and academic workloads (Brown et al., 2015). The pandemic may have intensified these stressors, placing student-athletes at a higher risk for poor mental health. For example, many athletes viewed the canceled sports season as a tragic loss (Schinke et al., 2020) that induced feelings of grief, frustration, and stress (National Collegiate Athletic Association Research, 2020; Schary and Lundqvist, 2021). McGuine et al. (2021) surveyed over 3,000 student-athletes in the US and found that athletes had 2.5 times higher depression scores during COVID-19 than athletes in a pre-COVID-19 group. Moreover, this increase was more pronounced for female athletes than male athletes. Another survey of US student-athletes reported that fear of COVID-19, feeling stressed, sad or depressed were emotional barriers to their training (National Collegiate Athletic Association Research, 2020). How mental health predicts motivation to train during a pandemic among Canadian student-athletes, and if there are gender differences, remains unclear.

### Motivation theory and research

Research shows that personal motivation is related to different levels of commitment to exercise (Ryan and Patrick, 2009). We used self-determination theory (SDT; Ryan and Deci, 2002) as our theoretical framework to understand how motivation could predict training behaviors among student-athletes because it has been extensively applied in the context of competitive sport (Ng et al., 2012; Clancy et al., 2016).

SDT distinguishes three types of motivation that exist along a continuum: intrinsic motivation, extrinsic motivation, and amotivation (Ryan and Deci, 2000). *Intrinsic motivation* is the most self-determined type of motivation, and involves engaging in an activity because of the pleasure and satisfaction of the activity itself. At the other end of the spectrum is *amotivation*, which involves a lack of engagement and motivation. Between these two are different levels of extrinsic motivation that range in their degree of internalization: *external motivation* (i.e., motivation is external, doing an activity for compliance or external rewards), *introjected motivation* (i.e., motivation is somewhat external, doing an activity out of guilt or feeling you ought to), *identified motivation* (i.e., motivation is somewhat internal, doing an activity because you value the goal), and *integrated motivation* (i.e., motivation is internal, doing an activity because it is important to your self-worth).

Motivational profiles can also be grouped as *autonomous motivation* and *controlled motivation* to reflect an individual's reasons for engaging in activities and their beliefs that the activities will satisfy their psychological needs (Ryan and Deci, 2000). Autonomous motivation (i.e., intrinsic, integrated, identified motivation) involves feeling self-directed and doing an activity because you find it interesting, satisfying, and it aligns with your sense of self. Controlled motivation (i.e., extrinsic and introjected motivation) involves external regulation and reflects doing behaviors for external rewards, to avoid punishment, or feeling guilty. Previous research showed that autonomous motivation helps individuals maintain long-term exercise goals (Teixeira et al., 2012; Willem et al., 2017).

Several studies have examined training motivation among athletes during the pandemic. Morbée et al. (2021) surveyed 207 cyclists in Belgium. While all the cyclists had difficulty motivating themselves to train, those who had autonomous motivation adapted to the changes and continued training whereas those who had controlled motivation or amotivation experienced difficulty. Likewise, Leyton-Román et al. (2021) surveyed 179 Spanish athletes and found that autonomous motivation and the basic psychological need of competence accounted for nearly 64% of the variance of an athlete's commitment to train. Also, Ruffault et al. (2020) surveyed 759 France athletes and found that participants who followed a training program during lockdown reported significantly lower anxiety and greater autonomous motivation than those who did not follow any training program. While the results suggest autonomous motivation was associated with motivation to train during the pandemic, the studies did not focus on student-athletes and data were collected between April and June 2020 when the return to sport was unclear.

### Purpose of the study

The impact of COVID-19 on the training among Canadian post-secondary student-athletes is unclear. It is also unclear how an athletes' mental health and motivational profile may have helped them adapt their training during the pandemic. Answers to these issues will provide information to coaches and trainers to better support student-athletes to adapt their training during the pandemic or an offseason. Using a correlational research design, we conducted a cross-sectional survey to address three research questions. First, we aimed to examine how athletes adapted their training and to identify what barriers they experienced to maintain their training. Second, we aimed to examine whether different motivation profiles were associated with differences in training behaviors and mental health. Based on theory and research, we developed four hypotheses. Hypothesis 1 (H1) stated that athletes who had higher autonomous motivation scores would report higher levels of training intensity. Hypothesis 2 (H2) stated that athletes who had higher controlled motivation or amotivation scores would report lower levels of training intensity. Hypothesis 3 (H3) stated that athletes who had higher autonomous motivation scores would report less mood disturbance. Hypothesis 4 (H4) stated that athletes who had higher controlled motivation or amotivation scores would report more mood disturbance. Third, we aimed to determine the extent to which access to training resources, mental health, and motivational profile predicted athletes' motivation to train.

## Materials and methods

### Participants

To be in our study, participants needed to be a post-secondary student who was a member of a sports team at a university or college in Canada. Thus, individuals playing recreational, intramural, or an individual-based sport (e.g., swimming, cross-country skiing) were excluded. Overall, 496 individuals participated in our survey. We removed 72 participants because they either completed <50% of the survey questions (*n* = 48), were not student-athletes (*n* = 15), or played an individual-based sport (*n* = 9). Our final sample consisted of 424 participants. Table 1 presents the demographic characteristics.

**Table 1:** Characteristics of the sample.

**Variable**	***n* (%)**
Age (*M, SD*)	19.77 (1.82)
**Gender**	
Male	125 (29.48)
Female	299 (70.52)
**Province of school**	
Alberta	151 (35.61)
British Columbia	69 (16.27)
Manitoba	22 (5.19)
New Brunswick	34 (8.02)
Newfoundland and Labrador	5 (1.18)
Nova Scotia	14 (3.30)
Ontario	99 (23.35)
Prince Edward Island	6 (1.42)
Quebec	22 (5.19)
Not reported	2 (0.47)
**Year entering**	
First	112 (26.42)
Second	115 (27.12)
Third	96 (22.64)
Fourth	67 (15.80)
Fifth	26 (6.13)
Not reported	8 (1.89)
**Sport Played**	
Basketball	87 (20.52)
Football	14 (3.30)
Hockey	87 (20.52)
Rugby	7 (1.65)
Soccer	73 (17.22)
Volleyball	156 (36.80)

### Measures

#### Demographics

Participants were asked demographic questions including gender, age, province of their school, year of study, and the sport they played.

#### Training and physical conditioning

This section of the survey consisted of eight questions. One question asked “*When do you think you will return to competing again*?” with response options ranging from 1 month to more than 6 months, as well as an unsure option. Two questions asked how many days a week they exercised during the off-season and currently during the pandemic. Five questions were adapted from Pillay et al.'s exercise maintenance survey tool (Pillay et al., 2020) that asked participants to report their current exercise routines. Of these questions, the first asked, “*At what intensity do you exercise?*” that was measured on a 3-point scale, with response options ranging from 1 (low) to 3 (high). The second asked, “*Have you reduced your training load and intensity during the pandemic?*” that was measured on a 4-point scale, with response options ranging from 1 (not at all) to 5 (a great deal). The third asked, “*How long do you exercise in a single session?*” and participants provided the average time in minutes. The fourth asked, “*Who do you train with?”* and participants could select options of exercising alone, directed digitally by a trainer, using technology like zoom with other athletes, or with a partner in-person. The fifth asked, “*What equipment do you use?* and participants indicated resources they had access to (e.g., sport-specific equipment, exercise machines).

#### Barriers to training

We developed a 12-item survey tool to assess participants perceived barriers to training, with nine items adapted from the Barriers to Athletics Training scale by the NCAA Research (National Collegiate Athletic Association Research, 2020). Items were assessed using a 5-point scale ranging from 1 (strongly disagree) to 5 (strongly agree). To identify the underlying factors of the survey, we conducted a principal components analysis with varimax rotation. We used three criteria to determine the number of factors: the a priori hypothesis that the survey was multidimensional, the eigenvalue-greater-than-one rule, and the interpretability of the factor solution. Before analysis, the variables were screened for normality and univariate outliers. The variables appeared normally distributed with no significant outliers. The analysis yielded a three-factor solution that provided the most clearly interpretable factor structure and accounted for 58.64% of the variance. We reviewed the items on each factor for conceptual clarity and labeled each factor. Factor 1 accounted for 22.06% of the variance and consisted of four items that related to having limited physical access to exercise facilities, specialized equipment, and trainers, so we labeled it *Limited Access to Training Resources*. Factor 2 accounted for 19.70% of the variance and consisted of four items that related to having difficulty maintaining a consistent training schedule, so we labeled it *Irregular Training Schedule*. Factor 3 accounted for 16.89% of the variance and consisted of four items that related to emotions such as fear of contracting COVID-19 and feeling stressed or depressed, so we labeled it *Emotional Distractions*. We calculated the mean subscale scores, with higher mean scores indicating a greater perceived barrier. The subscales showed good reliability with Cronbach alpha scores ranging from 0.66 to 0.83. The final scale is presented in Table 2.

**Table 2:** Factor loadings, means and standard deviations, and reliability scores for the Barriers to Training subscales.

	**Mean (*SD*)**	**Factor loadings**	**Cronbach alpha (α)**
**Factor 1: Limited Access to Training Resources**	**2.59 (1.00)**		0.83
I have access to appropriate facilities (R)	2.75 (1.25)	0.84	
I have access to appropriate equipment (R)	2.57 (1.22)	0.83	
I have access to a training partner (R)	2.55 (1.28)	0.74	
I have access to coaches or trainers (R)	2.48 (1.19)	0.75	
**Factor 2: Irregular Training Schedule**	**3.53 (0.85)**		0.73
The local regulations regarding travel and public gatherings have negatively impacted my training	3.63 (1.14)	0.64	
The intensity of my workouts has decreased	3.32 (1.19)	0.76	
I do not workout as long now	3.02 (1.19)	0.76	
My training has not been impacted at all by COVID-19 (R)	4.15 (1.04)	0.70	
**Factor 3: Emotional Distractions**	**2.73 (0.79)**		0.66
I fear being exposed to COVID-19	3.05 (1.19)	0.66	
I have more family and personal responsibilities that impact my training	3.24 (1.09)	0.53	
I am too stressed or anxious to train	2.38 (1.10)	0.81	
I am too sad or depressed to train	2.23 (1.10)	0.71	

(R) refers to reverse scored items.

#### Sports motivation

To assess athletes' motivation to train during the pandemic, we asked participants one question, “*I am motivated to train*” that was measured on a 5-point scale ranging from 1 (strongly disagree) to 5 (strongly agree). We assessed participant's motivation levels using the 18-item Sport Motivation Scale II (SMS-II; Pelletier et al., 2013). The SMS-II included six subscales to measure six types of motivation: intrinsic, integrated, identified, introjected, extrinsic, and amotivation. Participants' motivation was assessed using a 7-point Likert scale ranging from 1 (does not correspond at all) to 7 (corresponds completely). The subscales showed good reliability with Cronbach's alpha scores of 0.73 to 0.86. To be consistent with previous research (Leyton-Román et al., 2021), we calculated autonomous motivation as the mean of intrinsic, integrated, and identified motivation scores, and controlled motivation as the mean of extrinsic and introjected motivation.

#### Psychological wellbeing

We used the Profile of Mood States (POMS) questionnaire (Grove and Prapavessis, 1992) to assess psychological wellbeing. The POMS consisted of 40 adjectives such as depression, fatigue, and vigor. Each item was rated on a 5-point scale ranging from 0 (not at all) to 4 (extremely). We calculated a Total Mood Disturbance (TMD) score by summing the totals for the negative subscales (i.e., depression, fatigue, anger) and then subtracting the totals for the positive subscales (i.e., vigor, esteem-related affect). The survey showed good reliability with Cronbach's alpha scores of 0.66 to 0.95 (Grove and Prapavessis, 1992).

#### Procedure

Following institutional ethics approval (Pro00103321), we conducted an internet search for the social media groups (i.e., Facebook) of university and college athletic departments in Canada. This recruitment method was deemed most appropriate as social media is a common tool used by athletic departments to communicate with their athletes. Once a social media group was identified, we contacted the group administrator and asked to post an advertisement of our study on their website. When permission was granted, the study description and survey link was posted. Athletes who were interested in participating could read the study description and click a link that directed them to the survey website. From there, athletes read the consent form. Athletes indicated their consent to participate by clicking an “I consent to take this survey” button. The survey was conducted between August and September 2020, and took approximately 12 min to complete. All participants could choose to be entered in a draw for a $25 gift card to an online retailer.

#### Analytic strategy

We used SPSS to analyze the descriptive statistics, correlations, and *t*-tests and analysis of variances (ANOVA) to determine group differences. For H1 and H2, “training intensity” was defined using three questions: “*At what intensity do you exercise?,” “Have you reduced your training load and intensity during the pandemic?*,” and “*How long do you exercise in a single session?”* For H3 and H4, “mood disturbance” was defined as a high TMD score according to the POMS. For analyses comparing gender or year of study differences, we used the Welch's *t*-test and *F*-test. The Welch's test adjusts the degrees of freedom in the analysis to provide more reliable results when sample sizes and variances are unequal between groups (Delacre et al., 2017). Likewise, the Games-Howell *post-hoc* test was used for all follow-up analyses of group differences because it does not require equal sample sizes or assume homogeneity of variance (Delacre et al., 2019). For our third research question, we conducted a hierarchical multiple regression with the item “*I am motivated to train*” as the dependent variable and demographic variables age and gender, the three Barriers to Training subscales, motivational profiles, and TMD scores as predictors. Prior to the regression analysis, all variables were screened to ensure the assumptions were met. Inspecting the scatterplots showed that each predictor and dependent variable had a linear relationship, and examining the variables showed no significant outliers or multicollinearity. Therefore, we observed no violations in assumptions.

## Results

### Training routines

Table 3 presents the data on training and physical conditioning. Approximately a third believed they would return to competing within 3–6 months (*n* = 140, 33.0%) whereas another third was unsure (*n* = 133, 31.3%). Before COVID-19, nearly all participants (*n* = 399, 94.1%) exercised three or more days a week during the offseason, but this decreased during the pandemic (*n* = 319, 75.2%). During the pandemic, nearly 25% of athletes reported exercising 0–2 days per week (*n* = 105, 24.8%). When they did train, athletes reported training alone (*n* = 258, 60.9%). Exercise sessions were slightly over an hour long (*M* = 67.66, *SD* = 23.12), and most athletes reported exercising at a moderate intensity (*n* = 229, 54%). About 25% of participants did not have resistance bands, free weights, or sports-specific equipment to train at home, and 40% did not have a cardio machine. Across these variables, we observed no significant gender differences.

**Table 3:** Training and physical conditioning variables.

**Variables**	**All**	**Males**	**Females**
**When do you think you will return to competing again?**			
1 month of less	29 (6.84)	11 (8.80)	18 (6.02)
1–3 months	62 (14.62)	21 (16.80)	41 (13.71)
3–6 months	140 (33.02)	37 (29.602)	103 (34.45)
More than 6 months	60 (14.15)	15 (12.00)	45 (15.05)
Unsure	133 (31.37)	41 (32.80)	92 (30.77)
**Before COVID-19 how many days a week would you normally workout in an offseason?**			
0–2	25 (5.90)	6 (4.80)	19 (6.35)
3–4	183 (43.16)	46 (36.80)	137 (45.82)
5+	216 (50.94)	73 (58.40)	143 (47.83)
**How many days a week are you currently working out?**			
0–2	105 (24.76)	31 (24.80)	74 (24.80)
3–4	167 (39.39)	53 (42.40)	114 (38.13)
5+	152 (35.85)	41 (32.80)	111 (37.12)
**Who do you train with?**			
Alone	258 (60.85)	78 (62.40)	180 (60.20)
Digital with trainer	95 (22.41)	21 (16.80)	74 (24.75)
Zoom	56 (13.21)	10 (8.00)	46 (15.38)
With partner	189 (44.58)	50 (40.00)	139 (46.49)
**Have you reduced your training load and intensity?**			
None at all	66 (15.57)	26 (20.80)	40 (13.38)
A little	198 (46.70)	53 (42.40)	145 (48.49)
Unsure	15 (3.54)	4 (3.20)	11 (3.68)
A lot	115 (27.12)	32 (25.60)	83 (27.76)
A great deal	30 (7.08)	10 (8.00)	20 (6.69)
**At what intensity do you exercise?**			
Low	15 (3.54)	4 (3.20)	11 (3.68)
Moderate	229 (54.01)	72 (57.60)	157 (52.51)
High	180 (42.45)	49 (39.20)	131 (43.81)
**Do you have?**			
Sports specific equipment	323 (76.18)	94 (75.20)	229 (76.59)
Resistance bands	313 (73.82)	83 (66.40)	230 (76.92)
Free weights	319 (75.24)	96 (76.80)	223 (74.58)
Exercise machine (treadmill, stationary bike, etc)	255 (60.14)	68 (54.40)	187 (62.54)
Squat rack	193 (45.52)	60 (48.00)	133 (44.48)

### Barriers to training

The means and standard deviations for the three training barriers subscales are presented in Table 2. As shown, participants generally disagreed that they experienced difficulty accessing fitness resources or experienced emotional distractions. However, they generally agreed that they had difficulty maintaining a regular training schedule. We conducted three independent samples Welch's *t*-tests for each of the subscales to explore gender differences. First, Limited Access to Training Resources differed across genders, t_(236)_ = 1.96, *p* < 0.05, with males reporting more barriers to facilities and equipment (*M* = 2.73, *SD* = 0.99) than females (*M* = 2.53, *SD* = 1.00). There was also a gender difference for Emotional Distractions, t_(233)_ = −4.57, *p* < 0.001, with females reporting that emotional distractions created a barrier to training (*M* = 2.84, *SD* = 0.77) than males did (*M* = 2.46, *SD* = 0.77). There were no significant gender differences for Irregular Training Schedule, t_(217)_ = −0.65, *p* > 0.05.

### Motivational profiles

**Figure 1:**
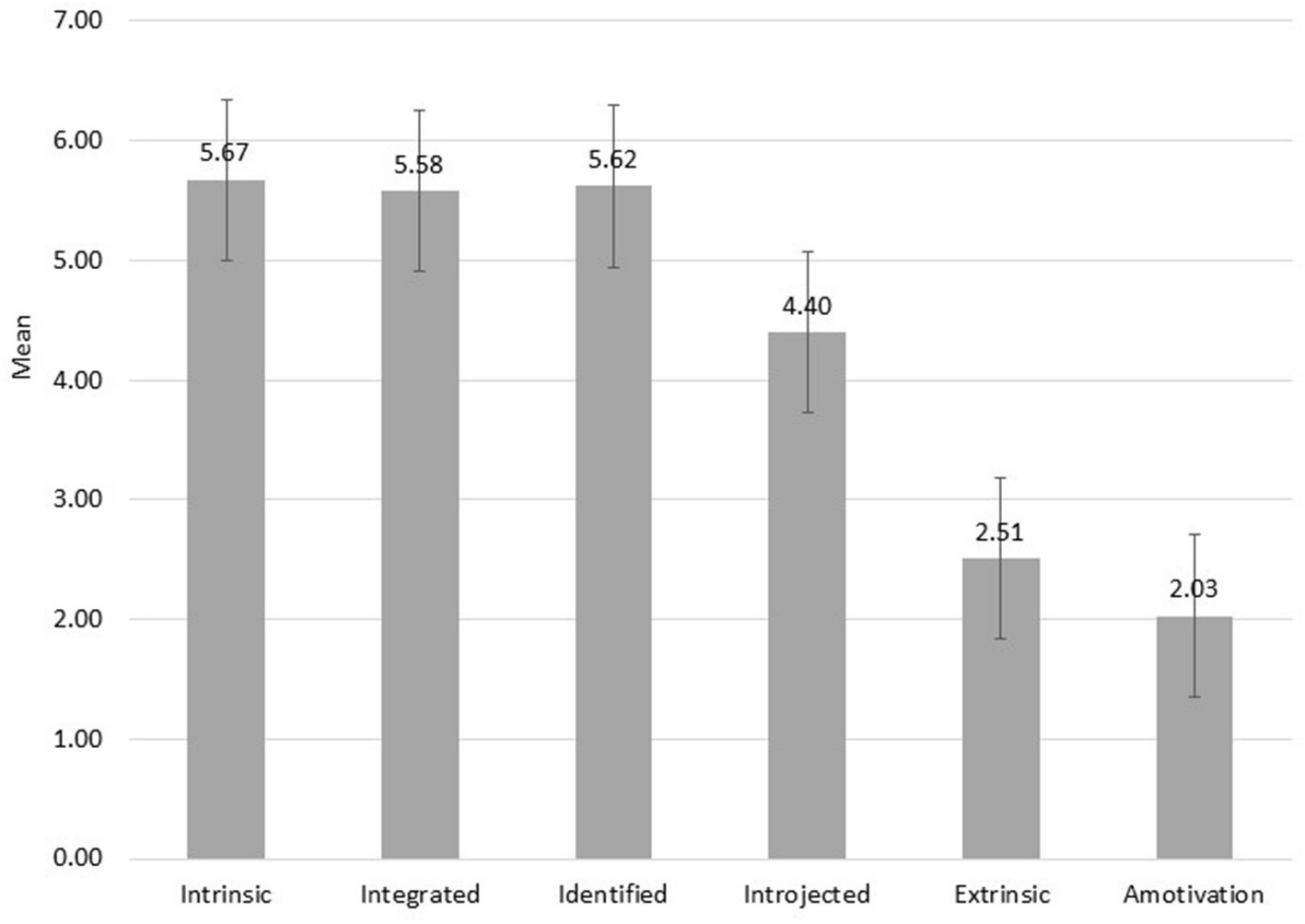
Motivation levels among the varsity athletes.

Overall, participants reported high levels of intrinsic, integrated, and identified motivation, moderate levels of introjected motivation, and low levels of extrinsic motivation and amotivation (Figure 1). We explored whether motivation levels differed by gender using a series of Welch's *t*-tests. Females reported higher levels of controlled motivation (*M* = 3.55, *SD* = 1.04) than males (*M* = 3.23, *SD* = 0.88), t_(272)_ = −3.02, *p* < 0.001. Exploring controlled motivation further, males reported higher intrinsic motivation (*M* = 5.81, *SD* = 1.09) than females (*M* = 5.61, *SD* = 1.12), t_(238)_ = 1.77, *p* < 0.05. Females reported higher levels of introjected motivation (*M* = 4.48, *SD* = 1.19) than males (*M* = 4.21, *SD* = 1.14), t_(242)_ = −2.20, *p* < 0.05, as well as higher levels of extrinsic motivation (*M* = 2.61, *SD* = 1.34) than males (*M* = 2.25, *SD* = 1.06), t_(291)_ = −2.96, *p* < 0.01. There were no gender differences for autonomous motivation.

We also examined year of study, and found that athletes differed in autonomous motivation, *F*_(4,132)_ = 2.34, *p* < 0.05. *Post hoc* comparisons showed second-year athletes reported higher autonomous motivation than third-year (*p* < 0.01). In contrast, third-year athletes reported higher amotivation than first-year athletes (*p* < 0.01) and second-year athletes (*p* < 0.05). When we explored autonomous motivation further, intrinsic motivation differed across years, *F*_(4,129)_ = 6.06, *p* < 001, whereby first-year athletes reported higher intrinsic motivation than third-year athletes (*p* < 0.01), and second-year athletes reported higher intrinsic motivation than third (*p* < 0.05) and fourth-year students (*p* < 0.05).

### Training and motivational profiles

We did not find support for H1, which stated that athletes with higher autonomous motivation scores would report greater levels of training intensity, but as Table 4 shows, athletes with higher levels of intrinsic motivation reported greater exercise intensity (*r* = 0.10, *p* < 0.05). We found partial support for H2, which stated that athletes who have higher controlled motivation or amotivation scores would report lower levels of training intensity. Athletes with higher levels of amotivation reported reducing their training load and intensity (*r* = 0.21, *p* < 0.01) and reducing minutes in a single exercise session (*r* = −0.10, *p* < 0.05). No other correlations were significant.

**Table 4:** Correlations among the training, mental health and motivation variables.

	**2**	**3**	**4**	**5**	**6**	**7**	**8**	**9**	**10**	**11**	**12**	**13**	**14**	**15**	**16**	**17**
1. Age	-0.10^*^	0.16^**^	0.11^*^	−0.07	−0.15^**^	−0.01	−0.12^*^	−0.05	0.07	−0.03	−0.09	−0.10^*^	0.10^*^	−0.05	0.13^**^	0.03
2. I have motivation to train		−0.32^**^	−0.44^**^	−0.38^**^	−0.37^**^	0.28^**^	−0.01	0.33^**^	0.18^**^	0.19^**^	0.09	−0.10^*^	−0.28^**^	0.25^**^	−0.39^**^	0.17^**^
3. Limited Access to Training Resources			0.39^**^	0.24^**^	0.21^**^	−0.09	−0.07	−0.08	−0.03	−0.11^*^	−0.06	−0.06	0.12^**^	−0.25^**^	0.40^**^	−0.12^*^
4. Irregular Training Routines				0.33^**^	0.39^**^	−0.02	0.11^*^	−0.10^*^	0.03	0.03	0.12^*^	0.05	0.22^**^	−0.45^**^	0.62^**^	−0.18^**^
5. Emotional Distractions					0.56^**^	−0.10^*^	0.24^**^	−0.20^**^	−0.02	−0.02	0.17^**^	0.22^**^	0.30^**^	−0.12^*^	0.20^*^	−0.09
6. Total Mood Disturbance						−0.02	0.28^**^	−0.10^*^	0.06	0.01	0.23^**^	0.23^**^	0.27^**^	−0.10^*^	0.23^*^	−0.10^*^
7. Autonomous Motivation							0.07	0.81^**^	0.81^**^	0.86^**^	0.30^**^	−0.16^**^	−0.41^**^	0.08	0.02	0.07
8. Controlled Motivation								−0.11^*^	−0.21^*^	0.09	0.80^**^	0.83^**^	0.32^**^	−0.03	0.04	−0.01
9. Intrinsic									0.44^**^	0.57^**^	0.12^*^	−0.28^**^	−0.42^**^	0.10^*^	−0.08	0.09
10. Integrated										0.56^**^	0.37^**^	−0.02	−0.27^**^	0.03	0.03	0.09
11. Identified											0.24^**^	−0.09	−0.31^**^	0.06	−0.01	−0.01
12. Introjected												0.33^**^	0.05	−0.07	0.21^**^	−0.10^*^
13. Extrinsic													0.46^**^	0.02	0.02	−0.10
14. Amotivated														0.07	0.21^**^	−0.10^*^
15. Training intensity															−0.44^**^	0.27^**^
16. Reduced training load																−0.19^**^
17. Exercise session length																

* p < 0.05,

** p < 0.01.

### Mental health and motivational profiles

Table 4 shows that autonomous motivation was not related to mental health, but athletes with higher levels of intrinsic motivation reported lower mood disturbance (*r* = −0.10, *p* < 0.05) which provided partial support for H3. We found support for H4, whereby higher ratings of mood disturbance were related to controlled motivation (*r* = 0.28, *p* < 0.01) and amotivation (*r* = *0.2*7, *p* < 0.01). We also explored gender differences on TMD scores using a Welch's *t-t*est and found a significant difference, t_(267)_ = −4.75, *p* < 0.001. Females reported more mood disturbance (*M* = 142.20, *SD* = 26.81) than males (*M* = 129.93, *SD* = 22.79).

### Predicting motivation to train

To determine what predicted an athletes' motivation to train during the pandemic, we conducted a hierarchical multiple regression with the item “*I am motivated to train*” as the dependent variable and four sets of predictor variables entered in steps. In step one, we entered gender and age to control for possible demographic differences. In step two, we entered the six motivation profiles, followed by the three Barriers to Training subscales in step three, and the TMD scores in step four.

As shown in Table 5, gender and age contributed significantly to the regression model, *F*_(2,410)_ = 10.36, *p* < 0.001 and accounted for 4.8% of the variance in motivation to train. Introducing the six motivation profiles explained an additional 11.8% of variance and was a significant change in variance, *F*_(6,404)_ = 9.51, *p* < 0.001. For step 3, adding the Barriers to Training subscales explained an additional 20.3% of variance and was a significant change in variance, *F*_(3,401)_ = 42.93, *p* < 0.001. In step four, adding TMD explained an additional 1.4% of variance yet was a significant change in variance, *F*_(1,400)_ = 8.81, *p* < 0.001. Examining the coefficients when all of the variables were entered into the model revealed that the significant predictors of motivation to train were being male, being younger, intrinsic and introjected motivation, having access to training resources, maintaining a regular training schedule, having fewer emotional distractions, and lower TMD. Overall, the model accounted for 38.3% of the variance.

**Table 5:** Hierarchical multiple regression for predicting motivation to train.

**Variable**	**β**	***t***	***R***	***R* ^2^**	**Δ*R*^2^**
**Step 1**			0.22	0.05	0.05^***^
Gender	−0.20	−4.02^***^			
Age	−0.15	−2.98^**^			
**Step 2**			0.41	0.17	0.12^***^
Gender	−0.17	−3.50^***^			
Age	−0.10	−2.13^*^			
Intrinsic	0.25	4.14^***^			
Integrated	0.03	0.45			
Identified	−0.03	−0.48			
Introjected	0.05	0.87			
Extrinsic	0.04	0.74			
Amotivation	−0.17	−3.02^**^			
**Step 3**			0.61	0.37	0.20^***^
Gender	−0.14	−3.21^**^			
Age	−0.08	−1.88			
Intrinsic	0.18	3.28^**^			
Integrated	0.05	0.97			
Identified	0.02	0.33			
Introjected	0.12	2.44^*^			
Extrinsic	−0.02	−0.34			
Amotivation	−0.02	−0.32			
Limited Training Resources	−0.13	−2.79^**^			
Irregular Training Routines	−0.31	−6.71^***^			
Emotional Distractions	−0.20	−4.36^***^			
**Step 4**			0.62	0.38	0.01^**^
Gender	−0.12	−2.95^**^			
Age	−0.10	−2.36^*^			
Intrinsic	0.18	3.43^***^			
Integrated	0.06	1.23			
Identified	0.01	0.21			
Introjected	0.12	2.64^**^			
Extrinsic	−0.01	−17			
Amotivation	−0.01	−0.03			
Limited Training Resources	−0.11	−2.63^**^			
Irregular Training Routines	−0.27	−5.85^***^			
Emotional Distractions	−0.14	−2.76^**^			
Total Mood Disturbance	−0.15	−2.97^**^			

* p < 0.05,

** p < 0.01,

*** p < 0.001.

## Discussion

Our findings described the ways in which student-athletes adapted their training, the barriers they encountered when attempting to train, their mental health, and the variables that predicted their motivation to train during the first 6 months of COVID-19 restrictions in Canada. Consistent with previous research on youth (National Collegiate Athletic Association Research, 2020; Pillay et al., 2020; McGuine et al., 2021; Shepherd et al., 2021), we found that student-athletes reduced their training loads and intensity, trained less frequently each week, and exercised for shorter periods of time. The decrease in training loads and intensity may be partly due to a lack of fitness resources available due to SD/L policies. That is, many fitness centers were closed to reduce the spread of COVID-19 and only about 60–75% of athletes had fitness equipment at home. Moreover, athletes tended to train alone, and the lack of sports-related interaction with teammates may have disrupted their motivation to engage in training. The abrupt ending of the sporting season and SD/L policies meant that student-athletes became isolated from each other and could no longer participate in activities that were central to their identity as athletes (Graupensperger et al., 2020; Shepherd et al., 2021).

Contrary to H1, autonomous motivation was not associated with greater training intensity. As most participants in our sample reported having high levels of autonomous motivation and believed that they would be returning to sport within 6 months or less, they may have generally continued to do activities (i.e., exercising) that they found inherently enjoyable. Indeed, previous research showed that individuals who were more intrinsically motivated to exercise were more likely to remain physically active (Deci and Ryan, 1985; Richard et al., 1997). In contrast, we found that student-athletes with higher levels of amotivation decreased their training load, training intensity, and exercise session length, which provided support for H2. As individuals with higher levels of amotivation have no desire to engage in a target behavior (Deci and Ryan, 1985), student-athletes high in amotivation may have struggled to maintain their training schedule on their own especially at a time when the return to sport was unknown.

Social isolation due to SD/L policies and quarantining can be a stressful experience for most people, and athletes may have experienced greater emotional distress as the structure, routine, and social support they often use as coping strategies for stress was severed (Usher, 2020). We found that athletes' motivational style was related to their mental wellbeing. Specifically, athletes higher in intrinsic motivation reported less mood disturbance, which provided partial support for H3, whereas athletes higher in controlled motivation and amotivation reported greater mood disturbance, which supported H4. While future research needs to investigate the underlying reasons for this difference, it is possible that exercise played a critical role in helping student-athletes cope during the pandemic. As previously mentioned, student-athletes high in autonomous motivation generally maintained their training and reported low mood disturbance. Previous research has shown that physical activity can improve mental health, and reduce depression and anxiety symptoms (Schuch and Stubbs, 2019), possibly as a result of providing mental distraction (Nicholls et al., 2015) or by releasing endorphins (Lubans et al., 2016). In the stressful times of SD/L policies due to COVID-19, the presence of intrinsic motivation may have been a protective factor as student-athletes focused on the satisfaction and pleasure of training and not the external rewards or approval.

Staying motivated to train during a prolonged offseason was more complex than simply not having access to training resources. Our regression analysis revealed that motivation to train was related to a unique set of predictors: being male, intrinsic motivation, introjected motivation, access to training resources, consistent training, and improved mental health. Compared to male athletes, female athletes reported poorer mental health and more emotional distractions that impacted their training. While these results are consistent with previous research (Håkansson et al., 2020; Leguizamo et al., 2021; Sanborn et al., 2021), the reason underlying these gender differences remains unclear and requires further investigation. Some researchers have suggested that females are motivated to exercise due to body-image and appearance concerns stemming from cultural pressures – an experience that males do not encounter (Frederick and Ryan, 1993). It is possible that SD/L policies and home confinement may have removed some of the social pressures females experience to maintain their physical appearance. It is also possible that gender differences reflect problems with self-reports. For example, Rice et al. (2020) suggested that male athletes are less likely to report mental health symptoms because doing so would be inconsistent with their perceptions of masculinity and strength.

Besides gender differences, experiencing disruptions to a training schedule was one of the greatest impediments to being motivated to train. As the rate of COVID-19 infections varied across Canada, and individuals may have needed to quarantine or follow stay-at-home protocols, training schedules may have been often interrupted. According to a large study of over 12,000 athletes across 146 countries, Washif et al. (2021) found that training interruptions during lockdown reduced motivation in over half of the athletes surveyed, and that athletes were usually exercising to promote general health and wellbeing rather than sport-specific skills because of a lack of resources. Indeed, the role of introjected motivation makes sense in this context as student-athletes were still a member of a sports team and therefore felt some obligation to maintain their training despite the lack of foreseeable external rewards for doing so.

While we could not locate a direct comparison to our study, some research has examined motivation among athletes during the pandemic (Ruffault et al., 2020; Leyton-Román et al., 2021; Morbée et al., 2021). For example, Morbée et al. (2021) examined cyclists and found that those who were more autonomously motivated were more likely to continue their training, and they experienced the highest level of need satisfaction and the lowest level of need frustration. Similarly, Leyton-Román et al. (2021) surveyed 179 athletes and found that autonomous motivation was a strong predictor of commitment to sports practice. In our study, we found that intrinsic motivation specifically was related to motivation to train. It is possible that intrinsic motivation is a protective factor that helped athletes adapt their training during the pandemic. For example, student-athletes high in intrinsic motivation may know that exercise satisfies their basic psychological needs and therefore choose these activities to fulfill their needs regardless of SD/L restrictions (Laporte et al., 2021). We also found that athletes high in amotivation were least adaptable, which was similar to Morbée et al. (2021). Previous research suggested these athletes may be at risk for exhaustion and burnout (Lonsdale and Hodge, 2011).

### Implications

Our findings have practical implications for coaching student-athletes during the pandemic or even extended offseason. Several researchers have recommended that coaches and trainers work with athletes to design training schedules that can be adapted for various levels of equipment access (Filiz et al., 2021). Moreover, Corcoran et al. (2020) explained how they recorded a series of exercise videos for their athletes to demonstrate proper technique and to establish appropriate training loads and intensity. These actions may help reduce significant reductions to training loads and help athletes maintain their physical conditioning and competitiveness (Gabbett, 2016; Andreato et al., 2020). However, it is the spikes in unsupervised training schedules – not simply the reductions in training loads – that put athletes at risk for non-contact, soft-tissue injury (Gabbett, 2016). We found that student-athletes struggled to maintain consistent training schedule during the pandemic. According to the Training-Injury Prevention Paradox model (Gabbett, 2016), non-contact injuries are more often a result of inappropriate training programs that involve excessive and rapid increases in training loads. Therefore, coaches should work with their athletes to develop training schedule that can be adapted to at-home training with limited equipment during the offseason.

Coaches and trainers could also have their student-athletes set training goals. Previous research shows that goal setting can reduce anxiety and increase motivation in sport (Hogue, 2020). Moreover, research shows that athletes benefit from social connectedness (Graupensperger et al., 2020; Filiz et al., 2021; Shepherd et al., 2021), so developing ways that teammates can connect remotely may be beneficial. As the motivational profile of an athlete may play a role in motivation to train, goal setting and regular team check-ins may be a way for athletes with higher levels of controlled motivation or amotivation to externally regulate the training behavior. As mental health professionals have suggested exercise can improve mental wellbeing during the pandemic (Brand et al., 2020) and reduce stress, anxiety, and depression (Nicholls et al., 2012), coaches could use several strategies to support their student-athletes.

### Limitations and future directions

Although our sample was large, our participants played a team-based sport. Thus, our results may not generalize to athletes who play individual sports such as swimming, golf, or track and field. Previous research showed that athletes who play team-based sports reported more anxious and depressive symptoms compared to athletes who played individual-based sports (Graupensperger et al., 2020; McGuine et al., 2021), but others have not found any differences (Di Fronso et al., 2022). While it is possible that COVID-19 may be more difficult for team-sport athletes as their social support networks were severed, future research should examine possible differences between athletes who play team-based sports vs. individual sports. Another limitation is that we conducted a cross-sectional study 6 months into the pandemic. As such, we are unable to assess how motivation or training schedules may have changed across time or what are the possible long-term effects. It is likely that the longer the crisis lasted, the more pronounced initially small differences among profiles may have become. Furthermore, our survey collected quantitative data that yielded a lot of responses but could not explore individual athletes' experiences the way a qualitative study might to help with understanding the impact the pandemic had on their mental health, training, and motivation.

## Conclusion

During the pandemic, athletes were forced into what may have felt like an extended offseason. Our results shed light on how student-athletes adapted their training, what training barriers they experienced, and how their mental health was impacted. One interesting finding was that student-athletes who were intrinsically motivated showed evidence of being able to adapt their training more easily than those higher in controlled motivation or amotivation. However, mental wellbeing and having an ability to maintain a regular training schedule were also key factors to maintaining motivation to train. Our findings can help coaches and trainers identify athletes who may need additional support and engagement to remain motivated to train during an offseason.

## Data availability statement

The raw data supporting the conclusions of this article will be made available by the authors, without undue reservation.

## Ethics statement

The studies involving human participants were reviewed and approved by Research Ethics Board 2 (REB2), Research Ethics Office, University of Alberta. The patients/participants provided their written informed consent to participate in this study.

## Author contributions

RP-S provided supervision over the project and wrote the current draft of the manuscript. RP-S and TZ contributed to conception and design of the study. TZ collected the data, helped organize the data, and assisted with some statistical analyses with the help of RP-S. RE assisted with editing and the updated literature review. All authors contributed to the revisions and approved the submitted version.

## Conflict of interest

The authors declare that the research was conducted in the absence of any commercial or financial relationships that could be construed as a potential conflict of interest.

## Publisher's note

All claims expressed in this article are solely those of the authors and do not necessarily represent those of their affiliated organizations, or those of the publisher, the editors and the reviewers. Any product that may be evaluated in this article, or claim that may be made by its manufacturer, is not guaranteed or endorsed by the publisher.
